# Solitary renal metastasis of esophageal squamous cell carcinoma mimicking primary renal neoplasm – A case report and literature review

**DOI:** 10.7603/s40681-016-0006-4

**Published:** 2016-02-23

**Authors:** Kai-Po Chang, Chi-Ping Huang, Han Chang

**Affiliations:** 1 Department of Pathology, China Medical University Hospital, 404 Taichung, Taiwan; 2 Department of Urology, China Medical University Hospital, 404 Taichung, Taiwan; 3 Department of Pathology, College of Medicine, China Medical University Hospital, 404 No. 2, Yuh-Der Road, Taichung, Taiwan

**Keywords:** Solitary renal metastasis, Esophageal cancer, Squamous cell carcinoma

## Abstract

Solitary renal metastasis of esophageal cancer is rare clinically, with only 14 cases being reported in the literature. The authors here report a case of a 53-year-old man with a metachronous hypopharyngeal and esophageal squamous cell carcinoma who developed a solitary renal metastasis after complete chemoradiotherapy for esophageal cancer, and subsequently received a left nephrectomy. The metastatic esophageal cancer was indistinguishable from primary renal neoplasm in the computed tomography but showed the histopathologic characteristic of esophageal cancer in directly invading the renal artery, and the tumor spreading in the kidney. The patient died of pneumonia two months after diagnosis. Among the previous 14 reported cases, 12 occurred in Asians, and their overall survival time ranges from two months to nine years after nephrectomy, either with or without adjuvant chemotherapy. Accordingly, a solitary renal mass in patients with a history of esophageal cancer is warranted to differentiate metastatic esophageal cancer from primary renal neoplasm, and a reliable therapy needs to be planned early for improving the patient’s chance of survival.

## 1. Introduction

Esophageal cancer is the sixth leading cause of cancer deaths worldwide, accounting for 406,800 cancer deaths in 2011 [1]. Most patients with esophageal cancer die from recurrence or metastasis, with the 5-year survival rate ranging from 15% to 25% [2]. The most common sites of metastasis are the liver, lungs, bones, and adrenal glands [3]. A unilateral renal metastasis is extremely rare. Herein, the authors report a case of metastatic esophageal squamous cell carcinoma to the kidney mimicking primary renal neoplasm in the computed tomography.

## 2. Case report

**Figure 1:**
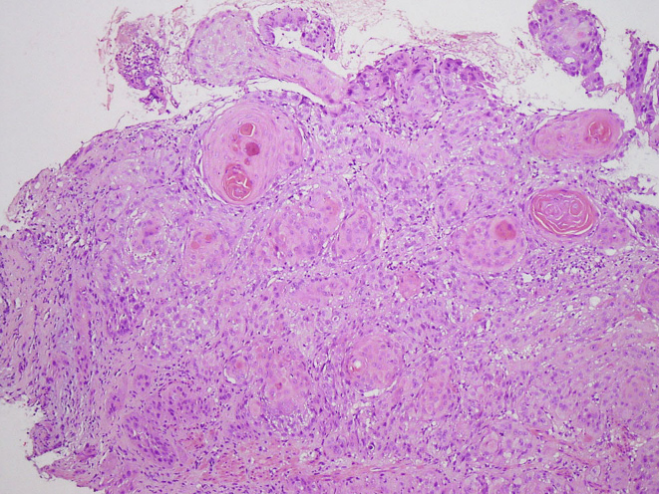

The 53-year-old Taiwanese man that is the subject of this case study had a history of hypopharyngeal squamous cell carcinoma that was histopathologically diagnosed in November, 2011. Complete remission was achieved after three cycles of concurrent radiotherapy and cisplatin-based chemotherapy. The patient remained asymptomatic during regular follow-ups in the otolaryngology clinic until January, 2013, when he started to have progressive dysphagia and dry throat. An esophagogastroduodenoscopy showed an ulcerative mass in the middle- to-lower third of his esophagus, and it was histopathologically diagnosed as keratinizing squamous cell carcinoma (Figure 1). Computed tomography (CT) showed this esophageal cancer displayed atrial wall invasion, and left paratracheal lymph node and lumbar spinal metastasis. The patient received concurrent radiotherapy and chemotherapy with 5-fluorouracil and cisplatin, with a partial response from the esophageal cancer, and neither tumor progression nor new metastasis in the follow-up positron emission tomography/CT and esophagogastroduodenoscopy.

**Figure 2:**
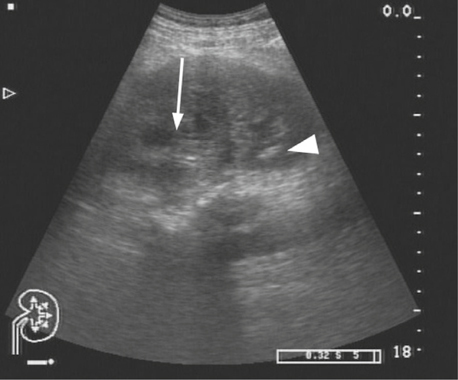

**Figure 3:**
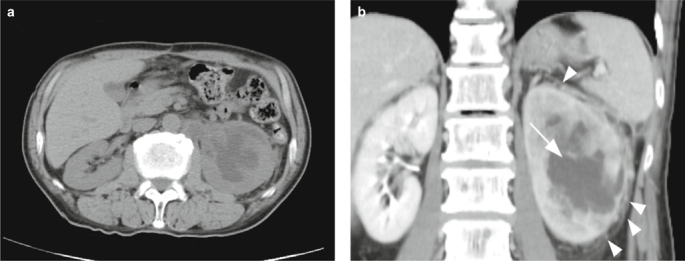

In June, 2014, the patient visited at the urology clinic complaining of intermittent gross hematuria and left flank pain for three weeks. Laboratory tests revealed renal insufficiency with a serum creatinine level of 1.75 mg/dl and glomerular filtration rate of 41 ml/min/1.73 m^2^. His blood urea nitrogen level was within a normal range. There was mild hyponatremia and normal serum levels of potassium and calcium. A renal sonography showed hydronephrosis and focal hyperechoic area of the left kidney (Figure 2). An abdominal CT showed a heterogeneously enhancing mass in the left kidney with local extensions to the peri-renal soft tissue and left adrenal gland (Figure 3). The outline of the renal pelvis and major calyces was preserved. Left nephrouretectomy was performed under suspicion of invasive urothelial carcinoma.

**Figure 4:**
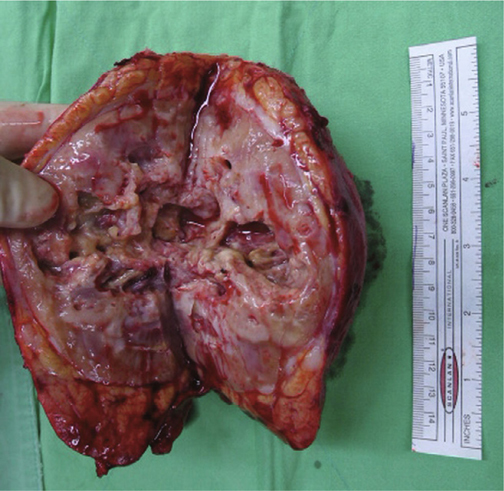

**Figure 5:**
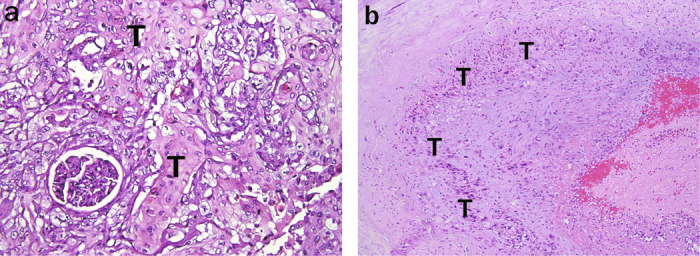

Macroscopically, the renal tumor measured 14 cm in its largest dimension, involved the renal pelvis and parenchyma, and was extended into the adrenal gland and perinephric fat (Figure 4). Microscopically, the renal tumor was composed of sheets or nests of polygonal cells with pleomorphic nuclei and markedly keratinizing cytoplasm (Figure 5a), which was identical to that of the previous esophageal cancer (Figure 1). The urothelium was inflamed without evidence of malignancy. The tumor invaded the adrenal gland and renal artery (Figure 5b), leading to focal renal infarct. The tumor showed immuno-positivity for cytokeratin (CK) 34βE12 and CK5/6, and negativity for CK7, CK20 and uroplakin III. Accordingly, this renal tumor was judged to be a metastatic esophageal squamous cell carcinoma. After surgery, the patient was sent to the intensive care unit for close observation. Aspiration pneumonia occurred and progressed to septic shock, and then the patient expired in November, 2014.

## 3. Discussion

Metastatic carcinoma of the kidney often appears as a solitary tumor or multiple subcapsular tumors infiltrating the renal parenchyma in CT [4]. Urothelial carcinoma of the renal pelvis, when in an advanced stage, also appears as an infiltrative tumor [5]. However, when the infiltrative tumor extensively involves the renal parenchyma, as in the present case, it may be difficult to differentiate a metastatic carcinoma from a urothelial carcinoma simply via image studies. In this situation, tumor immunohistochemistry and clinical information may be helpful in determining whether the tumor is metastatic or not [6, 7].

A previous autopsy study showed that 12-13% of esophagealcancer cases involve metastasis to the kidney [8, 9]. However, such cases are seldom encountered clinically. The present case and the 14 previously reported cases in the literature are briefly listed in Table 1 [3, 9-20]. All subjects were males. Three cases also had concomitant lung or brain metastases. Additionally, twelve cases were of Chinese, Korean, or Japanese ethnicity, and two were of French. Fourteen cases were squamous cell carcinomas and one was adenocarcinoma. The global cancer statistics show that esophageal squamous cell carcinoma is prevalent in Asia and adenocarcinoma in western countries [1, 21]. Moreover, esophageal squamous cell carcinoma generally has a poorer prognosis than adenocarcinoma [22]. Although cancer incidence and histologic type show a geographic difference, esophageal squamous cell carcinoma seems to have more of a tendency toward renal metastasis than esophageal adenocarcinoma.

The rarity of the detection of renal metastasis is discordant to the autopsy study. Because many patients with renal metastasis remains asymptomatic, Sun *et al*. [19] suggest that the asymptomatic manifestation may be attributable to an underestimation due to ignorance of the metastatic tumors. Our literature review shows that 6 of the 15 reported cases were asymptomatic and found incidentally, which is consistent with the view that renal metastasis of esophageal cancer is underestimated. Patients with metastatic disease could benefit from chemotherapy [23]. Therefore, routine screening of renal metastasis in patients with esophageal cancer may be necessary for early detection.

Histopathologic examination is commonly used to differentiate between primary and metastatic renal tumors. In the present case, the coexistence of the complete absence of urothelial precancerous lesions and the presence of arterial tumor thrombus was highly suggestive of a metastatic disease; in contrast, primary cancer cells usually invade small vessels and then follow a normal vascular flow pattern [22]. Although in routine hematoxylin and eosin stained slides, both metastatic and primary squamous cell carcinomas can be difficult to distinguish from urothelial carcinomas with extensive squamous differentiation, thorough sampling and careful examination are required in such cases to get clues as to the tumor cells’ origin. Immunohistochemical markers such as CK5/6, CK14, CK7, CK20, and uroplakin III have been reported and are potentially useful in distinguishing between urothelial carcinoma with squamous differentiation and squamous cell carcinoma [23-27]. The CK7/CK20 co-negative immunoprofile has been considered a characteristic for squamous cell carcinoma; however, urothelial carcinoma could also display this immunoprofile, particularly in the presence of extensive squamous differentiation [28, 29]. In the present case, there was a renal tumor with extensive squamous differentiation and immunoprofile of CK7/CK20/uroplakin III-negative and CK5/6-positive patterns, which are supportive of squamous cell carcinoma, but urothelial carcinoma cannot completely be ruled out with this information. Clinical information may be necessary in resolving this diagnostic difficulty.

**Table 1:** 

Case	Age/Ethnicity	Tumor type	Interval between metastasis and primary tumor	Survival time after renal metastasis
Pollack *et al*. 1987 [9]	62/NA	SCC	NA	2 months
Grise *et al*. 1987 [10]	56/French	SCC	24 months	6 months
(Two cases)	62/French	SCC	5 months	6 months
Kitami *et al*. 1987 [11]	61/Japanese	SCC	11 months	2 months
Nagai *et al*. 1989 [17]	50/Japanese	SCC	24 months	4 months
Shimizu *et al*. 1990 [18]	62/Japanese	SCC	5 months	NA
Miyoshi *et al*. 1997 [16]	57/Japanese	SCC	2 months	>24 months
(Two cases)	57/Japanese	SCC	12 months	2 months
Matsushita *et al*. 1998 [15]	74/Japanese	SCC	13 months	3 months
Mao *et al*. 2003 [20]	64/Chinese	SCC	36 months	>9 years
Lim *et al*. 2004 [14]	61/Korean	SCC	24 months	NA
Ku *et al*. 2005 [12]	65/Korean	SCC	21 months	>6 months
Lai *et al*. 2012 [13]	46/Chinese	SCC	24 months	24 months
Sun *et al*. 2014 [19]	64/Chinese	SCC	9 Months	3 months
The present case	53/Chinese	SCC	31 months	2 months

SCC, squamous cell carcinoma; ADC, adenocarcinoma; NA, not available.

A study by Nakagawa *et al*. has reported that the median survival rate is only 16 months if the hematologic metastasis of the esophageal carcinoma occurs [30]. Chemotherapy may improve the patient’s survival in such cases [23]. Yet it remains to be determined whether or not a nephrectomy is beneficial due to the rarity of cases. In the 15 cases of esophageal carcinomas with renal metastases, most patients received a nephrectomy, and 4 of the cases received concomitant adjuvant chemotherapy. Survival time after diagnosis was variable, ranging from 2 months to more than 9 years. Unfortunately, the efficacy of the adjuvant therapy cannot be concluded from the current reported cases.

## 4. Conclusions

A metastatic tumor should be considered in the differential diagnosis of a solitary renal mass in patients with a history of esophageal cancer, and a histopathological examination with the aid of immunohistochemistry is highly recommended for all suspected cases, since image study alone offers limited discrimination between metastatic tumors and primary renal neoplasms. An accurate diagnosis and early detection of a tumor metastasis are warranted for a reliable therapeutic plan.
